# A Wnt-Frz/Ror-Dsh Pathway Regulates Neurite Outgrowth in *Caenorhabditis elegans*

**DOI:** 10.1371/journal.pgen.1001056

**Published:** 2010-08-12

**Authors:** Song Song, Bo Zhang, Hui Sun, Xia Li, Yanhui Xiang, Zhonghua Liu, Xun Huang, Mei Ding

**Affiliations:** 1 Key Laboratory of Molecular and Developmental Biology, Institute of Genetics and Developmental Biology, Chinese Academy of Sciences, Beijing, China; 2 Graduate School, Chinese Academy of Sciences, Beijing, China; Harvard University, United States of America

## Abstract

One of the challenges to understand the organization of the nervous system has been to determine how axon guidance molecules govern axon outgrowth. Through an unbiased genetic screen, we identified a conserved Wnt pathway which is crucial for anterior-posterior (A/P) outgrowth of neurites from RME head motor neurons in *Caenorhabditis elegans*. The pathway is composed of the Wnt ligand CWN-2, the Frizzled receptors CFZ-2 and MIG-1, the co-receptor CAM-1/Ror, and the downstream component Dishevelled/DSH-1. Among these, CWN-2 acts as a local attractive cue for neurite outgrowth, and its activity can be partially substituted with other Wnts, suggesting that spatial distribution plays a role in the functional specificity of Wnts. As a co-receptor, CAM-1 functions cell-autonomously in neurons and, together with CFZ-2 and MIG-1, transmits the Wnt signal to downstream effectors. Yeast two-hybrid screening identified DSH-1 as a binding partner for CAM-1, indicating that CAM-1 could facilitate CWN-2/Wnt signaling by its physical association with DSH-1. Our study reveals an important role of a Wnt-Frz/Ror-Dsh pathway in regulating neurite A/P outgrowth.

## Author Summary

How do individual nerve fibers find their way along specific paths in a complex environment such as the developing central nervous system? A principal mechanism in axon guidance is binding of a receptor protein on the axon surface to a guidance molecule. However, it remains a mystery exactly how a limited number of guidance molecules can pilot the growth of billions of neurons. In this study, we used the nematode worm *Caenorhabditis elegans* to establish a system to follow the outgrowth of a single neurite. We then searched for genes affecting neurite development and uncovered a guidance molecule, CWN-2 (a member of the well-known Wnt family of signaling proteins), which attracts neurite growth. We also identified two Wnt receptors (CFZ-2 and MIG-1, from the Frizzled family), a co-receptor (CAM-1, from the Ror family), and an effector (DSH-1, from the Dsh family). Together these proteins convey the Wnt guidance signal into the neurite and influence its growth. In addition, our study reveals that the restricted spatial localization of the Wnt signal, together with the specific combination of Wnt receptors and effectors expressed in the neurite, are important for the complex function of guidance molecules.

## Introduction

During development of the nervous system, an individual neuron needs to be guided to its proper target through interactions with environmental guidance cues. Studies during the past twenty years have demonstrated that the ligand-receptor guidance mechanisms essential for this process are broadly present throughout the animal kingdom including worms, flies, and mice [1], [2].

Based on the body axis, the guidance action can be subdivided into Dorsal-Ventral (D/V) guidance and Anterior-Posterior (A/P) guidance. The well-defined D/V guidance cues include Netrin, Slit, Semaphorin and Ephrin [3]. It is generally accepted that through their receptors, these guidance signals trigger downstream events, which are less well characterized, and finally act on the cytoskeleton to modulate the extension and/or turning of the growth cone at the tip of axon [4].

Wnt signaling, first identified in regulating embryonic pattern formation, has recently been linked to neurite A/P guidance [5]. Binding of Wnt to the extracellular CRD domain of the Frizzled receptor (Frz) activates Dishevelled (Dsh). Once Dsh is activated, downstream signaling can take three different paths [6]. In the canonical pathway, when Wnt is present, Dsh acts through GSK3β and Axin to stabilize β-catenin, which then translocates from the cytoplasm to the nucleus, thus turning on gene transcription. In the calcium pathway, Dsh regulates calcium signaling including protein kinase C (PKC) and Ca^2+^/calmodulin-dependent protein kinase II (CaMKII), in events such as conversion extension during embryonic development. Dsh can also function through the small GTPases Rho/Rac, subsequently activating downstream JNK kinase to modulate the actin cytoskeleton in the establishment of planar cell polarity (also known as the PCP pathway). However, exactly how Wnt ligands and other components of the Wnt pathways regulate axon A/P guidance is not well understood.

In the spinal cord of mice, Wnt4 is present in the floor plate and attracts commissural axons through interaction with its receptor Frz3 [7]. PKCζ, atypical protein kinase C, and phosphatidylinositol-3-kinases (PI3K) are required for Wnt-mediated axon A/P outgrowth [8]. In mice, Wnt5 and Wnt1 gradients were found to be important for the repulsion of cortico-spinal axons through RyK [9]. In *Drosophila*, Wnt5 repels anterior commissural growth cones that express *Derailed* (*drl*), an atypical RyK gene [10]. These findings pinpoint the essential role of the Wnt pathway in A/P axon guidance, but also raise many more questions. Do all Wnts and their receptors contribute to A/P guidance? Since Wnt pathway components are widely present throughout the nervous system, how is specificity achieved for an individual neuron, or a subset of neurons, in A/P guidance? What is the difference between the Wnts and/or their receptors? Can they substitute each other functionally?

One way to answer these questions is to combine unbiased genetic screens and suitable molecular manipulations to systematically investigate the molecular mechanisms underlying axon A/P guidance. The *Caenorhabditis elegans* genome contains five *Wnts*, and some of them have been studied in great detail, including *egl-20*, *lin-44* and *mom-2* [11]–[13]. Besides embryonic/larval tissue patterning, several Wnts have been shown to function in nervous system development, including neuron migration, polarity, neurite extension, neurite pruning, synaptogenesis and, most recently, nerve ring organization [14]–[19]. However, the exact roles of Wnts and related components during axon A/P outgrowth are less well characterized.

Here we describe the usage of a pair of *C. elegans* head motor neurons to systematically investigate the regulatory machinery of neurite A/P outgrowth. From the genetic screen, we recovered a set of Wnt components and, specifically, we found that CWN-2/Wnt could function locally as an attractive cue and act on DSH-1 through the Frz receptors CFZ-2 and MIG-1, and the co-receptor CAM-1/Ror, to guide neurite outgrowth. In addition, the activity of CWN-2 can be partially substituted by expressing other Wnts locally, indicating that part of the functional specificity of Wnts is through tissue-specific gene expression. Together, our study reveals a specific Wnt-Frz/Ror-Dsh pathway regulating A/P neurite guidance in *C. elegans*.

## Results

### A genetic program regulating the neurite growth of RMED/V neurons

**Figure 1:**
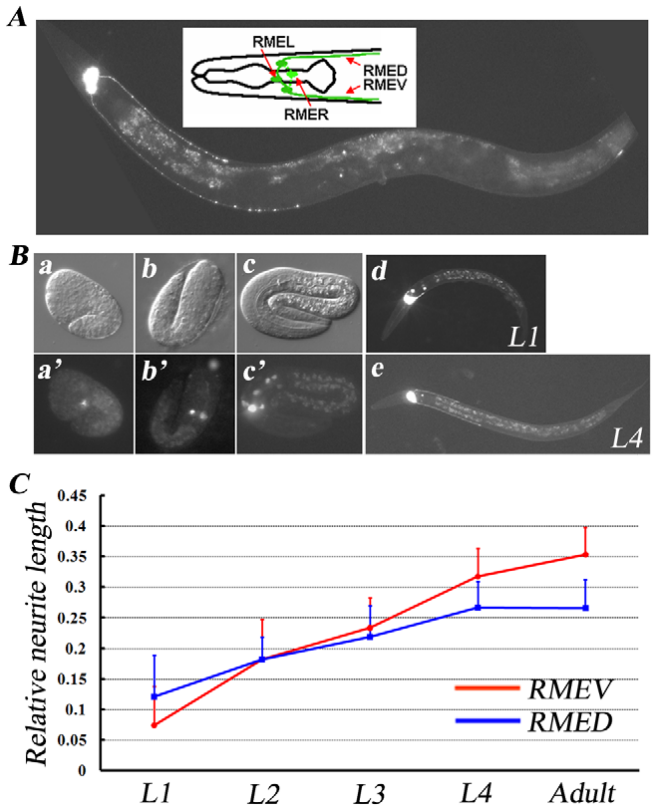
GABAergic RME neurons. (A) A GFP fluorescence image of an *unc-30(ju54);juIs76* animal. *juIs76* (*Punc-25::GFP*) highlights the RME GABAergic neurons in *unc-30(ju54)* mutant animals. The insert shows a schematic drawing of all four RME neurons. Anterior is to the left and dorsal is up. (B) RME neurons at different developmental stages, including a: comma stage; b: two-fold stage; c: three-fold stage, viewed with the *juIs76* marker. a, b, and c are DIC images; a′, b′, and c′ are fluorescence images. RMED/V neurons start sending out their posterior processes during the late embryonic stage (c and c′), and continue to grow posteriorly during larval stages (d and e). (C) The relative length of RMED and REMV posterior neurites at different developmental stages. Relative neurite length is defined as the ratio of neurite length to body length. Error bars represent the standard error of the mean (SEM).

The RME neurons are a set of four GABAergic motor neurons that innervate head muscles and regulate foraging movements [20]. The members of this class have an unusual four-fold symmetry; cell bodies are situated mid-dorsally (RMED), mid-ventrally (RMEV), left laterally (RMEL) and right laterally (RMER). In addition to the processes round the nerve ring, RMED and RMEV each sends out an extra process, which runs down the dorsal and ventral cords respectively and then terminates around the middle of the body (Figure 1A). Previously, we reported that in the absence of transcription factor AHR-1, the RMEL and RMER cells adopt RMED or RMEV cell fates and send out posterior processes, indicating that the outgrowth of posterior processes is genetically programmed [21].

Using a GFP transgene (*juIs76*) driven by the GABAergic neuron-specific promoter *Punc-25* [22], we were able to visualize the morphology of RME neurons in *unc-30* mutant animals. *unc-30* encodes a homeodomain protein that controls the specification of type D GABA neurons [23]. In the absence of UNC-30, *unc-25* expression in type D neurons is abolished [24]; however its expression remains unchanged in RME neurons (Figure 1A). Therefore, using the *juIs76* marker in the *unc-30* mutant background, we were able to follow the development of RME neurons in living animals. The RME cell bodies were first visualized around the bean/comma stage during embryogenesis (Figure 1B). By the L1 stage, the posterior extending processes could be identified unambiguously. We measured the relative length of the RMED/V posterior extending processes as the ratio to the body length and found that the relative length continues increasing and reaches a plateau in the adult stage (Figure 1C). We also noticed that individual variation in terms of the relative RMED/V lengths is rather small. By the adult stage, RMEV processes stop at a certain distance away from the vulva, at which time the length of the RMEV process is on average ∼35% of body length, while the RMED process is about ∼26% of body length (Figure 1C). Therefore, for rest of this paper, all phenotypes are scored at the young adult stage in *unc-30* mutant background.

**Figure 2:**
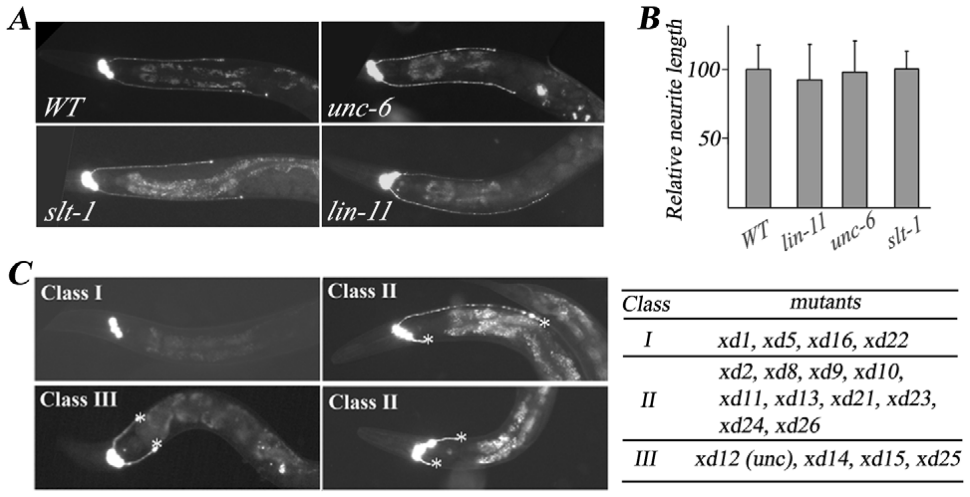
Mutants with RMED/V neurite outgrowth defect isolated from the genetic screen. (A) RME neuron fluorescence images of animals with different genetic backgrounds. *unc-30(ju54);juIs76* is treated as wild type (WT). RMED/V posterior neurite outgrowth is unaffected in the D/V guidance cue mutants *unc-6(ev400)* and *slt-1(eh15)* and the vulvaless mutant *lin-11(n389)*. (B) Quantification analysis of mutant phenotypes shown in (A). The average relative neurite length in wild type is set as 100. Error bars represent SEM. (C) Phenotypes and list of the three classes of mutants isolated from the genetic screen. Most Class I mutant animals lack both RMED and RMEV processes. Class II mutants display both shortened and normal length of neurites. In Class III mutants, both RMED and RMEV processes are short. Asterisk indicates the end of the process.

To uncover the genetic program that regulates RMED/V neurite outgrowth, we first tested whether classical axon guidance molecules are involved. When we introduced the GFP marker into *unc-6* and *slt-1* mutants [25], [26], we found that neither *unc-6(ev400)* nor *slt-1(eh15)* affects the growth of RMED/V A-P processes, indicating that the signals for RMED/V neurite growth are different from commissural axon guidance signals (Figure 2A and 2B). Since in wild type, neither the RMED nor the RMEV process can extend beyond the vulva, we asked whether the vulva serves as a physical boundary preventing neurite growth. When we examined the length of RMED/V processes in *lin-11*, a mutant with no vulva [27], we found there is no detectable deficit in the length of RMED/V neurites, suggesting that the vulva does not play a role in RME A/P growth (Figure 2A and 2B).

To systemically dissect the guidance signals required for RMED/V neurons, we performed a genetic screen for mutants with altered RMED/V neurite length. This yielded a total of 18 mutants, the phenotypes of which can be divided into three classes (Figure 2C). Class I mutants (*xd1*, *xd5*, *xd16*, and *xd22*) lack both RMED and RMEV processes. Class II mutants (*xd2*, *xd8*, *xd9*, *xd10*, *xd11*, *xd13*, *xd21*, *xd23*, *xd24*, and *xd26*) exhibit a variable phenotype including lack of processes, short processes and processes of normal length. In Class III mutants (*xd12*, *xd14*, *xd15*, and *xd25*), both RMED and RMEV display short processes and the phenotype is almost 100% penetrant. To reveal the molecular mechanism underlying RMED/V neurite growth, several of the genes were cloned and the detailed functional analysis is reported in following sections.

### *cwn-2/Wnt* regulates neurite A/P growth

**Figure 3:**
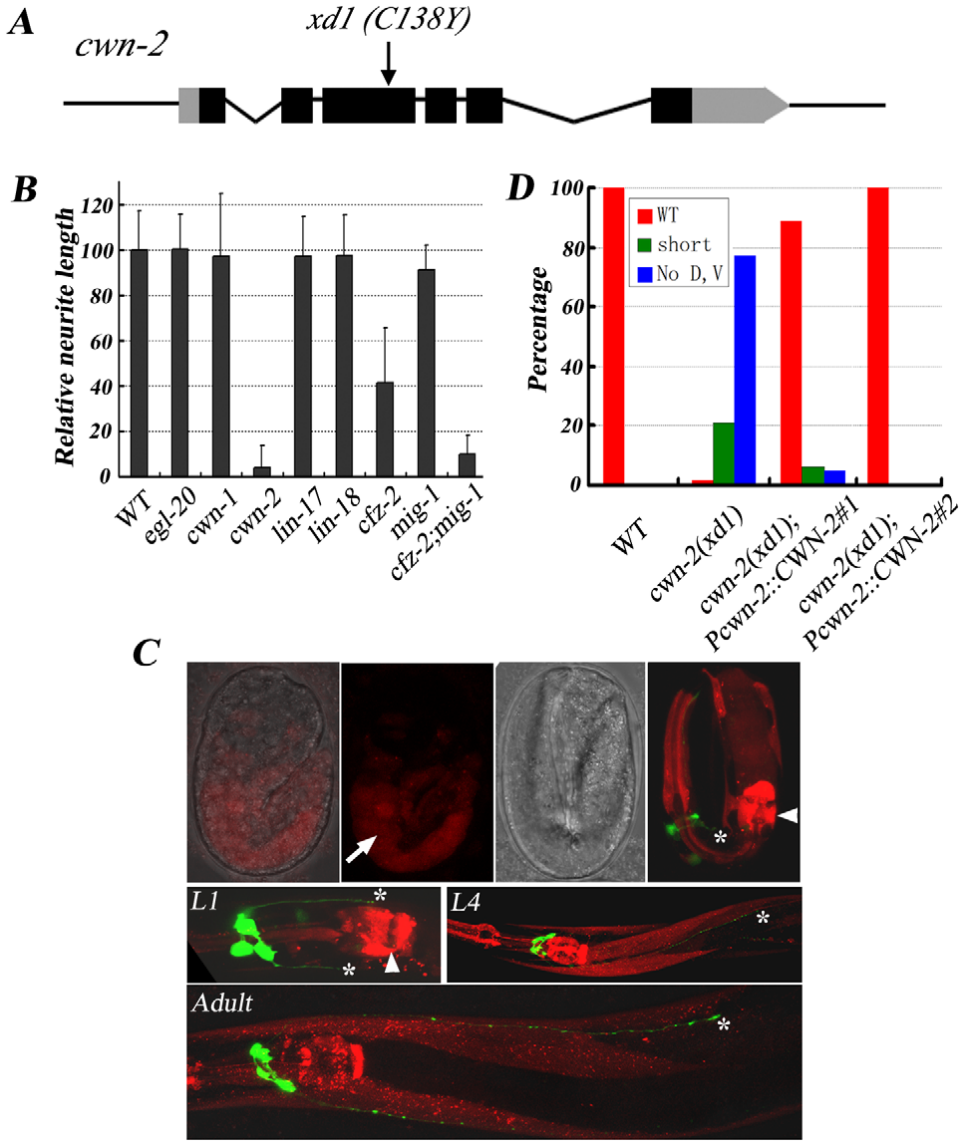
*cwn-2* regulates RMED/V neurite A/P outgrowth. (A) *xd1* is a C138Y missense mutation in the *cwn-2* locus. Black boxes are exons and grey boxes are UTRs. (B) Phenotypic quantification of RMED neurite A/P outgrowth defect in Wnt pathway receptor and ligand mutants. The average relative neurite length in wild type (*unc-30;juIs76*) is set as 100. Error bars represent SEM. Note that *mig-1(e1787);cfz-2(ok1201)* double mutants mimic *cwn-2(xd1)*. (C) *Pcwn-2::mCherry* expression pattern (Red) in different developmental stages. Green is RMED/V neurons highlighted by *juIs76* marker. Top panels: fluorescence and bright field images of embryos with *Pcwn-2::mCherry*. In a 2-fold stage embryo, *cwn-2* is mainly expressed in the intestine (arrow). The highest level of mCherry signal is observed in the posterior pharyngeal bulb and the pharyngeal-intestine valve before hatching (arrowhead). After L1 stage, *Pcwn-2::mCherry* is expressed in the pharynx, body wall muscles and some ventral cord neurons. Asterisks point to the tips of RMED/V neurites. (D) Quantification analysis of the rescue activity of the *cwn-2* genomic fragment in *cwn-2(xd1)*. The length of both RMED and RMEV were compared to wild-type controls (*unc-30;juIs76*). The extent of RMED and RMEV extension was classified into three categories: “WT” stands for wild-type length in both RMED and RMEV neurites; “no D, V” stands for absence of both RMED and RMEV neurites (Class I phenotype); and “short” indicates an intermediate phenotype between “WT” and “No D, V” (including shorter processes and absence of either RMED or RMEV process). Results from two independent transgenic lines are presented.

In animals carrying the class I mutation *xd1*, both RMED and RMEV lose posterior neurites. In addition, there are short anterior processes growing out from RMED/V cells with low frequency. These phenotypes remain in aged animals, indicating that the deficiency is not due to the delayed neurite outgrowth. Genetic mapping, transgenic rescue and complementation analysis identified *xd1* as an allele of *cwn-2*. *cwn-2* encodes one of the five Wnts in *C. elegans*. Genomic DNA sequencing revealed a G to A mutation in *xd1*, resulting in replacement of a conserved cysteine by tyrosine (Figure 3A). The lack of RMED/V processes in *cwn-2* mutants could be due to failure of neurite outgrowth or alternatively to a cell fate change, such as RMED/V cells adopting the RMEL/R cell fate. To distinguish these two possibilities, we examined *lim-6* reporter expression pattern in *cwn-2* mutants. In wild type, *lim-6* is specifically expressed in RMEL/R cells but not in RMED/V cells [28]. If RMED/V cells adopt the RMEL/R cell fate, *lim-6* should be expressed in RMED/V. In fact, we found that *lim-6* expression was still restricted to RMEL/R cells in *cwn-2* mutants (data not shown). These data suggested that CWN-2/Wnt likely directly regulates RME neurite A/P growth with no significant alteration of cell fate. To test whether *cwn-2* affects neurite A/P growth in other neurons, we examined the AVE neuron with a *Popt-3::mCherry* marker. Similar to RMEV, the AVE neuron has a posterior process along the ventral nerve cord, which terminates before the vulva region [29]. We found that the posterior growth of the AVE neurite is not affected in *cwn-2* mutants (data not shown), suggesting that *cwn-2* specifically regulates RMED/V neurite posterior extension.

There are five *Wnts* (*cwn-1*, *cwn-2*, *egl-20*, *lin-44*, and *mom-2*) in the *C. elegans* genome. To test whether other Wnts are involved in RMED/V outgrowth, we examined mutants of *cwn-1*, *egl-20*, *lin-44*, and *mom-2*. None of these mutants exhibits defects in RME neurite A/P growth (Figure 3B and data not shown). We then generated *lin-44;cwn-1* and *lin-44;egl-20* double mutants and found that they do not display any detectable outgrowth defect either (data not shown). Although we can't exclude the possibility that other Wnts, in particular *mom-2*, which is maternally required for viability, may play a rather minor role, above data suggest that among all five Wnt ligands, *cwn-2* likely plays a major role in RMED/V neurite outgrowth.

### Wnt functions locally to attract neurite outgrowth

Of five Wnts, why is CWN-2 so important for RME A/P outgrowth? One explanation is that the spatial localization of CWN-2 determines its function specificity. We analyzed the expression pattern of all Wnts using a *promoter::mCherry* fusion assay. Consistent with previous reports [16], [17], [30], *egl-20* and *lin-44* are mainly expressed in the posterior and vulval region. *mom-2* and *cwn-1* have low levels of expression in the head region and high levels of expression in the posterior body (Figure S1). *Pcwn-2::mCherry* is visible in the intestine and part of the pharyngeal region during the embryonic stage. After hatching, *cwn-2* is expressed in the anterior part of the worm, including pharynx, body wall muscle and ventral cord neurons (Figure 3C). *cwn-2* promoter-driven *cwn-2* can fully rescue the *cwn-2* mutant defects, suggesting that the *Pcwn-2::mCherry* pattern may represent *cwn-2* expression in vivo (Figure 3D and Figure S2).

**Figure 4:**
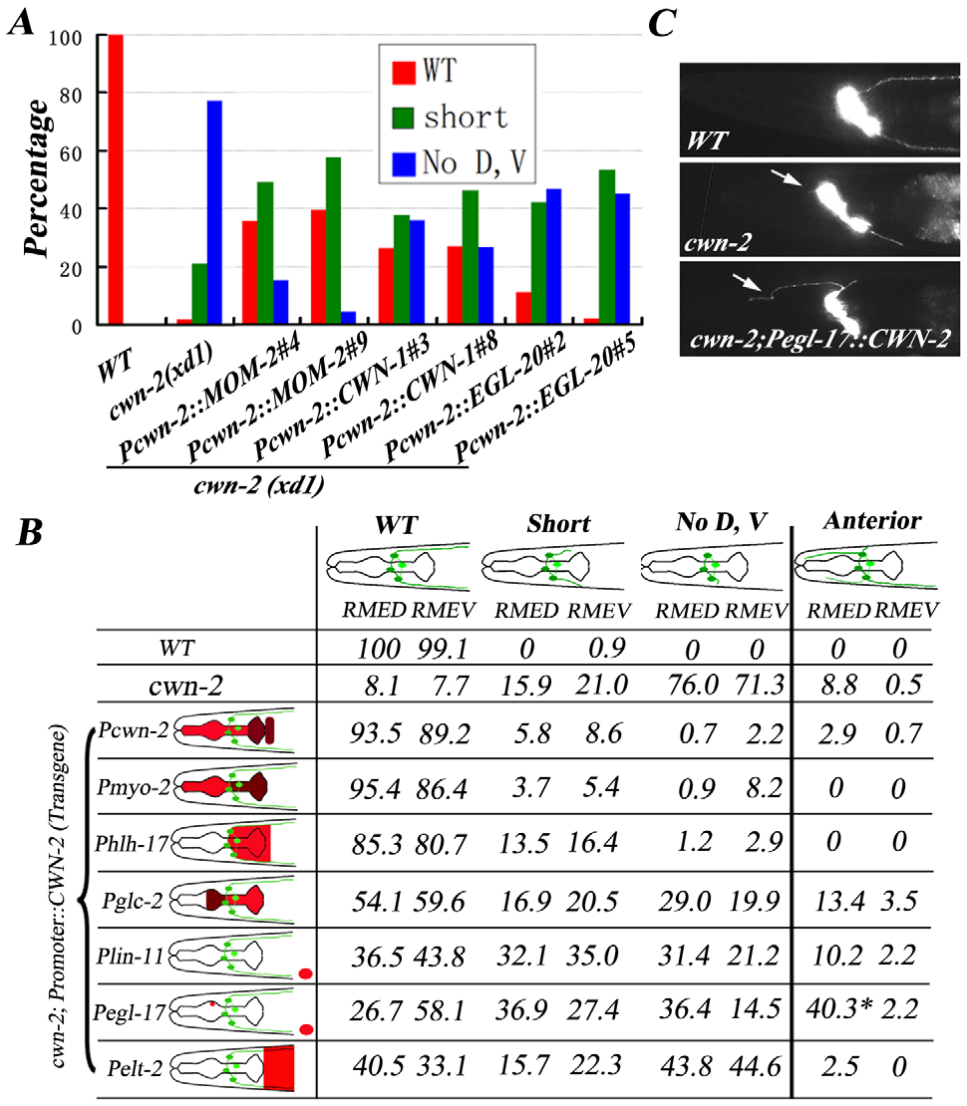
CWN-2 functions as an attractive cue. (A) Quantification analysis showing that other *Wnts* when under control of the *cwn-2* promoter can partially rescue *cwn-2(xd1)* mutant phenotype. Results from two independent transgenic lines are presented. The DNA injection concentration is 20 ng/µl. (B) CWN-2 expression, driven by different promoters, causes different levels of rescuing activity. Posterior extension and anterior growth of RMED and RMEV were evaluated separately. Schematic diagrams of the different posterior and anterior neurite phenotypes are shown at the top. The classification of the posterior phenotypes is the same as for the rescue assays in Figure 3. The color coding in the schematic drawing of the head (left) represents the expression patterns of different promoters. The expression level is relatively higher in the brown regions than in the red regions based on transcriptional mCherry assay. For the *lin-11* and *egl-17* promoters, the distance from the vulva (indicated by a red oval) to the head is not proportional. Expression of CWN-2 under control of the *egl-17* promoter is able to trigger RMED anterior neurite extension (*). (C) Phenotype of anterior extension (arrows) in wild type, *cwn-2(xd1)* mutants and *Pegl-17::CWN-2*-rescued *cwn-2(xd1)* mutants.

We wondered whether other Wnts could substitute CWN-2 when expressed in the same position as CWN-2. We introduced *cwn-1*, *mom-2*, *egl-20*, or *lin-44* cDNA driven by the *cwn-2* promoter into *cwn-2* null mutants and found that all of them could rescue the *cwn-2* mutant phenotype to some degree (Figure 4A and Figure S3), suggesting the functional specificity of a particular Wnt could, at least, partially due to whether it is present locally and the enforced high level of other Wnts may overcome the intrinsic difference among Wnts.

Next, we asked whether CWN-2/Wnt play an attractive or repulsive role in guiding RMED/V neurite outgrowth. We utilized tissue-specific promoters to express *cwn-2* in various places around the nerve ring region and examined the correlation between the direction of neurite outgrowth and the position of the *cwn-2* gene product in *cwn-2* mutant background (Figure 4B). Most transgenic lines express CWN-2 in a comparable level to wild type and *Pcwn-2::CWN-2* transgenes by Q-RT-PCR (Figure S4). We firstly expressed *cwn-2* in the whole pharynx using the *myo-2* promoter. Similar to the *cwn-2* promoter, *myo-2* has a slightly higher expression level in the posterior region of the nerve ring than the anterior region. We found that the *myo-2* promoter achieved full rescue activity. In contrast, when we expressed *cwn-2* under control of the *glc-2* promoter, which exhibits a higher level of expression in the anterior region of the nerve ring than the posterior region, we observed less rescuing activity. In addition, we found a considerably increased number of animals with RMED/V neurites that extended in the anterior direction. When *cwn-2* is expressed only in the posterior region of the nerve ring with the *hlh-17* promoter, the majority of *cwn-2* mutants regain the wild-type-like RMED/V neurite growth pattern. Above data suggests that CWN-2 may act as an attractive cue to guide RMED/V A/P neurite growth. In consistence with this hypothesis, when we expressed CWN-2 more posteriorly towards the vulva region using the *lin-11* promoter, we found that it also exhibited partial rescue activity. Interestingly, when *cwn-2* is expressed under control of the *egl-17* promoter, which drives expression in the vulva as well as dorsal M4 neurons in the pharynx [31], we found, in addition to weak rescuing activity, many more RMED neurons projecting neurites towards the anterior (Figure 4B). These data suggest that the presence of CWN-2 in the anterior dorsal region of the pharynx could redirect neurites to grow towards anterior (Figure 4B and 4C). Then could this rerouting phenotype be suppressed by CWN-2 from its normal source? We performed the same experiment in wild-type animals and found that the anterior outgrowth phenotype in the *Pegl-17::CWN-2* expressing animals could be fully suppressed (Figure S2), suggesting that CWN-2 functions as an attractive cue and CWN-2 molecules from different locations could compete each other to direct neurite outgrowth.

If CWN-2 functions indeed as an attractive cue, one would expect that CWN-2 should present at the posterior region of RMED/V neurite tips during process extension. Therefore, we closely monitored the *Pcwn-2::mCherry* expression at different developmental stages. The mCherry signal first appeared in the intestine before the expression of *Punc-25::GFP* (Figure 3C) during embryogenesis. At late embryonic stage when RMED/V neurite start extending posteriorly, the highest expression of CWN-2 is found at posterior pharyngneal bulb and the pharyngeal-intestinal valve (Figure 3C). When animals are at L1 stage, the RMED/V neurites grow towards the posterior pharynx, where CWN-2 displays relatively higher expression level (Figure 3C). The expression pattern of CWN-2 at both embryonic and L1 stages correlates well with the attractant role of CWN-2 during neurite outgrowth. After L1 stage, the neurites continue their growth and pass the posterior pharynx; meanwhile CWN-2 is also found expressed in body muscles and some ventral cord neurons (Figure 3C). It is possible the continuous growth of RMED/V neurites in the later larval stage relies on other guidance cues. Alternatively, the temporary experience with CWN-2 attractant during embryonic and L1 stages could be sufficient to maintain the extension of RMED/V processes. While we can not rule out other mechanisms which may work together with CWN-2 to guide neurite growth, current evidences support a notion that CWN-2 acts as an attractive cue to regulate RMED/V neurite A/P outgrowth.

### CAM-1 may act as a receptor for CWN-2

**Figure 5:**
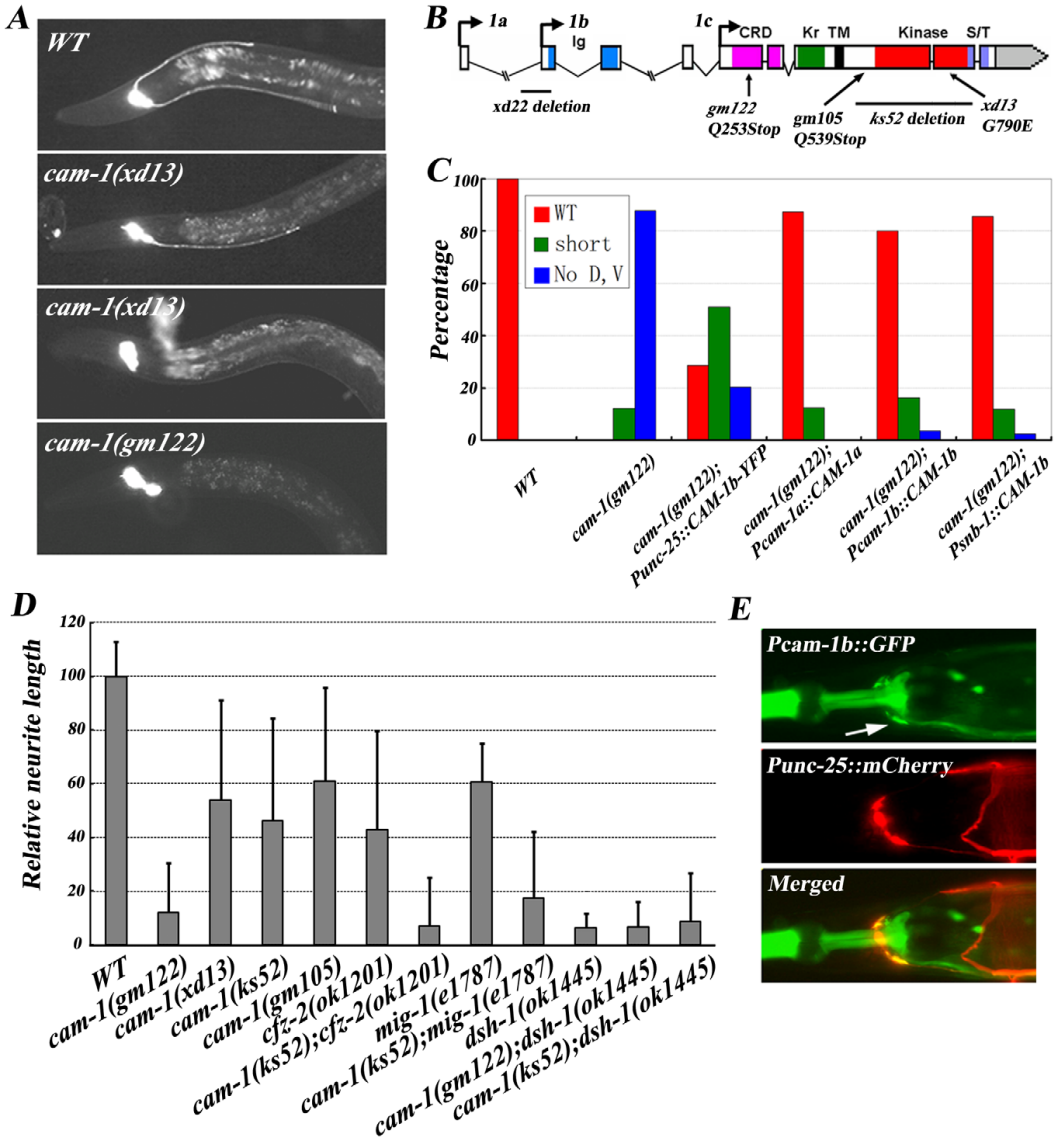
*cam-1* regulates RMED/V neurite A/P guidance. (A) RME neuron fluorescence images of animals with different genetic backgrounds. The *cam-1* partial loss-of-function mutant *xd13* shows a variable phenotype and the null mutant *gm122* has a *cwn-2* like phenotype. (B) Molecular lesions in the *cam-1* mutants *xd22*, *gm122*, *gm105*, *ks52* and *xd13*. The color-coded domain structure of *cam-1* is shown. Ig, immunoglobulin domain; CRD, cysteine rich domain; Kr, kringle domain; TM, transmembrane domain; Kinase, kinase domain; S/T, serine and theronine-rich domain. Three *cam-1* isoforms are indicated. (C) Quantification of the rescuing activity of *cam-1* transgenes. (D) Quantification of the RMEV neurite A/P outgrowth defect in different mutants. *cam-1* functions synergistically with *cfz-2* and *mig-1*. The average relative neurite length in wild type is set as 100. Error bars represent SEM. (E) *cam-1* is expressed in RME neurons (arrow).

From the genetic screen, we identified two alleles of *cam-1*, *xd22* (class I) and *xd13* (class II) (Figure 5A). *cam-1* encodes the *C. elegans* homolog of the CRD domain-containing receptor tyrosine kinase Ror2 [32], [33]. There are three isoforms of *cam-1*. *cam-1a* is the longest, while *cam-1b* lacks exon 1 and *cam-1c* lacks exons 1, 2, 3 and 4 (Figure 5B). In addition to the protein length, the transcription regulatory elements are different among these isoforms. Sequencing results showed that *xd13* causes a glycine to glutamic acid change in the kinase domain while *xd22* is a 607bp in-frame deletion, which deletes the start codon of the *cam-1b* isoform and part of the Ig domain (Figure 5B). When wild-type *cam-1* transgene was introduced into null mutants, full rescue activity was observed, indicating that *cam-1* is indeed required for RMED/V neurite growth (Figure 5C). A putative null allele of *cam-1(gm122)* results in a complete lack of RMED/V posterior neurites, mimicking the class I *xd22* mutant phenotype (Figure 5A). Thus, *xd22* is likely a null while *xd13* is a partial loss-of-function mutation. Indeed, loss of the intracellular domain of CAM-1 (*ks52* and *gm105* allele, for instance) only causes partial loss of function (Figure 5B and 5D). Furthermore, CAM-1 without the intracellular kinase domain retains only partial rescuing activity, while in the absence of the extracellular CRD domains, CAM-1 loses its function completely (Figure S2 and data not shown). This suggests that kinase activity is important but not essential for CAM-1 function.

In mammals, binding of Wnt5a to Ror protein stimulates its kinase activity [34]. The extracellular domain of CAM-1 could physically interact with EGL-20/Wnt in vitro [35]. However, it was initially suggested that CAM-1 did not act as a Wnt receptor in *C. elegans* but instead functioned as a Wnt or Frizzled antagonist during cell migration and other biological processes [36]. Recently, CAM-1 was identified as a receptor for CWN-2 in regulating nerve ring organization [16]. Thus, the effect of CAM-1 on RMED/V neurite growth could be explained by CAM-1 acting as a receptor for CWN-2, or, alternatively, CAM-1 may sequester other Wnts and therefore facilitate CWN-2 function indirectly. To distinguish between these two possibilities, we performed the following experiments. First, we tested whether CAM-1 is expressed in the CWN-2 responsive cells, which are the RMED and RMEV neurons. We identified *cam-1*-expressing cells by expressing GFP under the control of the *cam-1a* and *cam-1b* promoter. To visualize RME cells, *Punc-25::mCherry* was co-injected into worms. In the nerve ring region, *unc-25* is only expressed in the four RME cells. It was evident that both *Pcam-1a* and *Pcam-1b* drove GFP expression in the nervous system. Specifically, *Pcam-1a::GFP* highlights RMED and RMEV cells, while *Pcam-1b::GFP* is present in all four RME cells (Figure 5E and data not shown).

Next, we asked whether *cam-1* functions within *cwn-2* responsive cells. When we expressed *cam-1* in RME cells using the *unc-25* promoter, we found that it could rescue the *cam-1* mutant phenotype (Figure 5C). The rescue activity of *Pcam-1a::CAM-1a* is also consistent with the role of *cam-1* in RMED and RMEV cells specifically. However, we noticed that expressing *cam-1* in the whole nervous system using the *snb-1* promoter achieved better rescue than expressing *cam-1* in RME cells alone (Figure 5C). Therefore, to address whether CAM-1 activity is needed in non-CWN-2-responsive cells, we used the *unc-86* or *unc-4* promoter to express *cam-1* in neurons excluding RME cells and found that neither promoter exhibits rescuing activity (Figure S5). Similarly, we used the *hlh-17* promoter to express *cam-1* in cephalic sheath glia cells, which are near the nerve ring, but this could not alleviate the mutant phenotype either (Figure S5). We also examined whether additional expression of *cam-1* in non-RME cells could facilitate *cam-1* function. Co-injecting *Punc-4::CAM-1b* could not enhance *Punc-25::CAM-1b-YFP* rescuing activity, indicating that *cam-1* function is not required in non-RME cells (Figure S5). In addition, we found that the over-expression of *cwn-2* could not suppress *cam-1* null mutants (Figure S2), implying that the *cam-1* may function downstream of *cwn-2*. Taken together, these data suggest that CAM-1 likely serves as a CWN-2 receptor on RMED/V cells to regulate RMED/V neurite growth.

### *cam-1* interacts with *cfz-2* and *mig-1* genetically

There are four Frz receptors (CFZ-2, LIN-17, MIG-1, and MOM-5) and one atypical receptor tyrosine kinase (Ryk) receptor (LIN-18) in *C. elegans* [11]. Besides CAM-1, are any of these receptors involved in mediating the CWN-2 signal in RME neurite A/P outgrowth? Because none of the class I mutants turns out to be allelic to the Wnt receptors mentioned above, we reasoned that Wnt receptor mutants may display relatively weak (class II) phenotypes due to gene redundancy. Indeed, *cfz-2* displays a variable phenotype, while *mig-1* has a very mild defect in RMED/V neurite growth (Figure 3B). Moreover, the *mig-1;cfz-2* double mutant phenotype mimics class I mutants, indicating that CFZ-2 and MIG-1 function redundantly as Wnt receptors in mediating the CWN-2 signal (Figure 3B). In contrast, the other three Wnt receptors, LIN-17, LIN-18 and MOM-*5*, appear not to play a role in RMED/V neurite outgrowth (Figure 3B and data not shown).

What is the relationship between Frz receptors (CFZ-2 and MIG-1) and CAM-1? Compared to *cam-1* null mutants, null mutants of *cfz-2* or *mig-1* only display weak phenotypes and *mig-1;cfz-2* double mutants exhibit a strong phenotype resembling *cam-1* null mutants. This suggests that CAM-1 could act as the main receptor for CWN-2/Wnt, while CFZ-2 and MIG-1 are two co-receptors for CAM-1. Consistent with this idea, the double mutants *cam-1(weak);cfz-2(null)* and *mig-1(null)*; *cam-1(weak)* all exhibit phenotypes resembling *cam-1* full loss-of-function mutants (Figure 5D).

### A conserved Wnt pathway including DSH-1 functions within RMED/V to regulate neurite outgrowth

**Figure 6:**
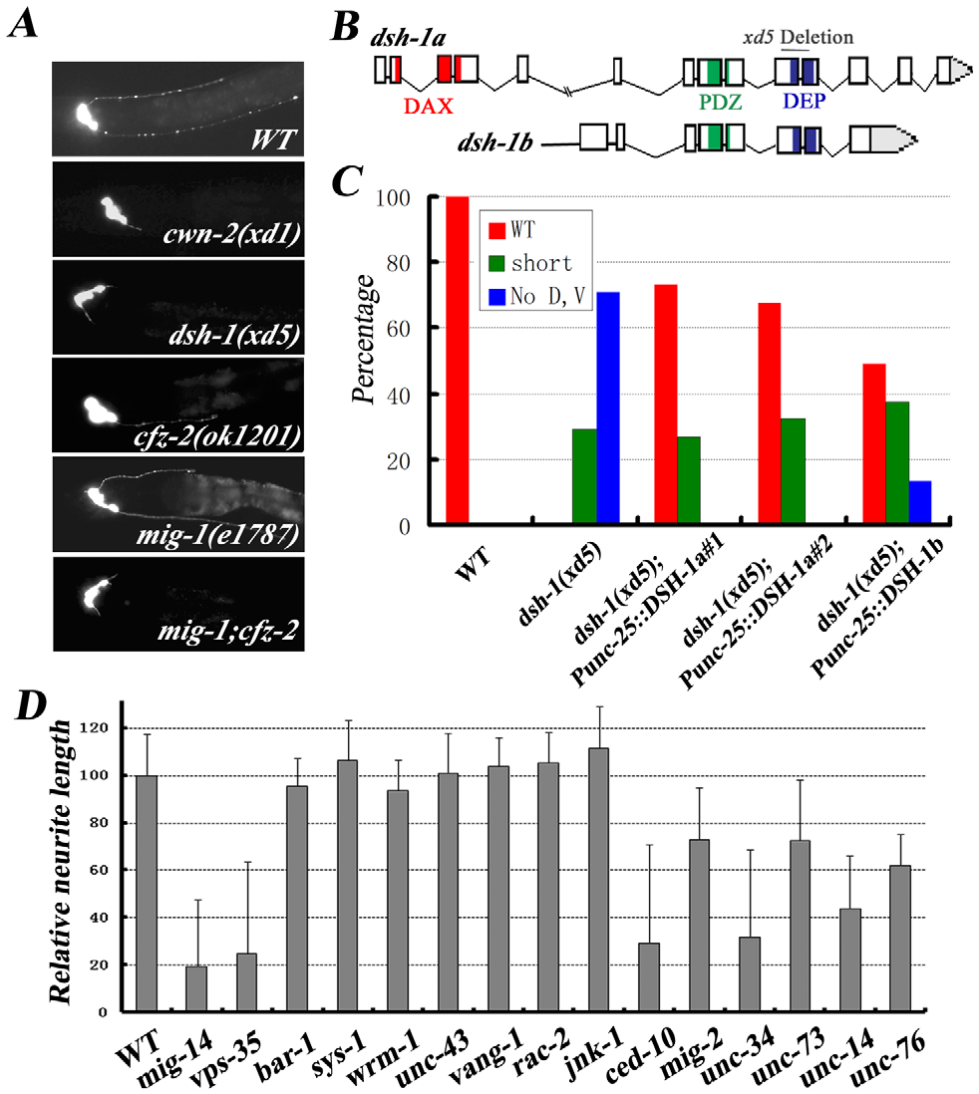
DSH-1 and a conserved Wnt pathway regulate RMED/V neurite outgrowth. (A) Fluorescence images of RMED/V neurons in various Wnt mutant animals. *cwn-2(xd1)*, *dsh-1(xd5)* and *mig-1(e1787);cfz-2(ok1201)* double mutant exhibit the same phenotype. (B) Molecular lesion in the *dsh-1* mutant *xd5*. The domain structure of *dsh-1* is shown. DAX: domain present in Dishevelled and axin; PDZ: PSD-95, Dlg, and ZO-1/2 domain; DEP: Dishevelled, Egl-10, and Pleckstrin domain. Two DSH-1 isoforms are indicated. *xd5* causes a small deletion. (C) Quantification of the rescuing activity of *dsh-1* transgenes. Expression of *dsh-1* in RMED/V neurons can rescue *dsh-1(xd5)* mutant phenotype. (D) Quantification of the RMED neurite A/P outgrowth defect in different mutants, including components of the three different downstream pathways of Wnt and factors affecting the actin-microtubulin cytoskeleton.

The above results indicate that CWN-2 activates its membrane receptors (CAM-1, CFZ-2 and MIG-1) on RME cells then triggers downstream events to regulate RME neurite A/P outgrowth. What are the downstream events? The *xd5* mutation, which causes loss of both RMED and RMEV processes, turns out to be an allele of *dsh-1* (Figure 6A). Dishevelled (Dsh) is a cytoplasmic multi-domain protein that is required for all known branches of the Wnt signaling pathway [6]. There are two isoforms of *dsh-1*: *dsh-1a* and *dsh-1b*. Compared to DSH-1a, DSH-1b lacks the N-terminal DAX domain, which is essential for canonical Wnt signaling. The *xd5* mutation is a 254bp deletion that affects both isoforms and results in a stop codon in the DEP domain (Figure 6B). Therefore, it is a putative null allele of *dsh-1*. In agreement with this, the null mutant of *dsh-1(ok1445)* displays the same phenotype as *dsh-1(xd5)* (Figure 5D). Moreover, using the *unc-25* promoter to drive *dsh-1a* expression in RMED/V, we observed full rescuing activity, suggesting that *dsh-1* function is required in RMED/V neurons (Figure 6C and Figure S2).

What are the downstream signals after DSH-1? There are three proposed signal pathways: the canonical pathway, the calcium pathway and the PCP pathway. In the canonical Wnt pathway, DSH acts through β-catenin to regulate gene transcription. However, we found that none of the canonical pathway components including BAR-1, PRY-1, WRM-1 and the newly identified β-catenin SYS-1, appears to have a role in RMED/V neurite A/P growth (Figure 6D and data not shown). In addition, in the absence of the calcium pathway component CamKII (UNC-43), the growth of RMED/V neurites is indistinguishable from wild type (Figure 6D). We then tested whether the PCP pathway is involved. In the PCP pathway, DSH activates the Rho/Rac GTPases, thus regulating cytoskeleton organization. Although the absence of MIG-2/Rho or CED-10/Rac does cause RMED/V neurite outgrowth defects, *rac-2*, *vang-1*, or *jnk-1* mutant does not display any visible phenotype. Therefore, whether PCP pathway is involved in RMED/V neurite outgrowth remains to be determined (Figure 6D). In addition, we found that mutants with deficits in cytoskeleton organization (such as *unc-34/enable*, *unc-73/Rho-GEF*, *unc-76*, and *unc-14*) also display shortened axon phenotype and the axon re-routing phenotype in *cwn-2;Pegl-17::CWN-2* animals could be partially suppressed by these mutations, consistent with the general role of cytoskeleton re-arrangement during neurite outgrowth (Figure 6D and Figure S2).

Previous reports show that Wnt gradient formation requires the secretion machinery in Wnt-producing cells [37], [38]. Similar to *cwn-2* and *dsh-1*, mutants of *mig-14* and *vps-35*, which act upstream to regulate the secretion of Wnts, display a class I phenotype (Figure 6D), suggesting that retromer complex and other secretion components are involved in RME neurite A/P outgrowth.

### CAM-1 interacts with DSH-1 physically

**Figure 7:**
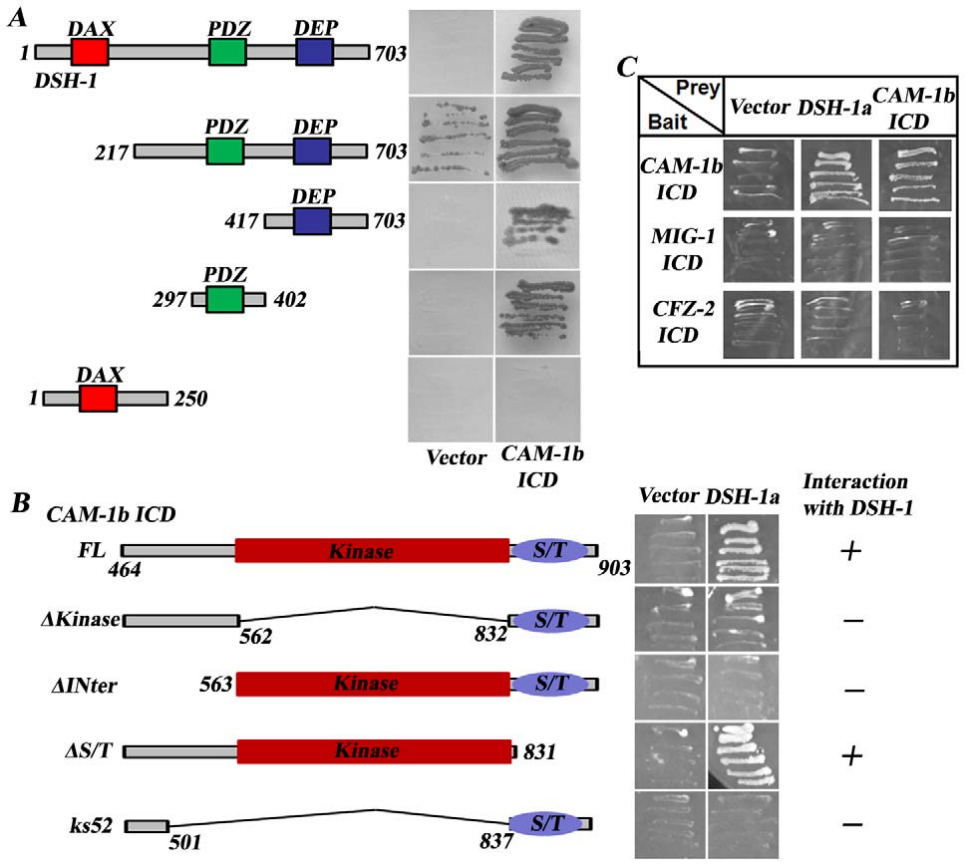
The CAM-1 intracellular domain interacts with DSH-1. (A) The PDZ and DEP domains are important for the binding of DSH-1 to CAM-1 intracellular domain (ICD). Empty vector (Vector) was used as a control. (B) The kinase domain and the region before the kinase domain are crucial for DSH-1 interacting. (C) MIG-1 ICD and CFZ-2 ICD do not interact with CAM-1 or DSH-1.

To further explore how CAM-1 transmits CWN-2 signals to downstream components, we performed a yeast two-hybrid screen using the CAM-1 intracellular domain as bait. From the screen, we identified that the full length DSH-1 could bind to CAM-1 (Figure 7A). We further narrowed down the binding activity of DSH-1 to its PDZ and DEP domains, while the DAX domain is not required for CAM-1 binding (Figure 7A). Furthermore, we made truncated CAM-1 intracellular domain constructs and found that the kinase domain and the junction region between kinase and transmembrane domain are important for DSH-1 binding (Figure 7B). We also tested whether there is any physical interaction between DSH-1 and other Wnt receptors. The results demonstrated that neither CFZ-2 nor MIG-1 intracellular domain could bind DSH-1 in yeast two-hybrid assay (Figure 7C).

The DAX domain of DSH has been shown to bind to Axin and is important for the canonical Wnt signaling pathway [39]. Through alternative splicing, two DSH-1 isoforms (DSH-1a and DSH-1b) exist in *C. elegans*; DSH-1b lacks the DAX domain (Figure 6B). We found that *Pdsh-1b::mCherry* but not *Pdsh-1a::mCherry* is expressed in RME cells (data not shown). Moreover, DSH-1b alone significantly rescues the *dsh-1* null phenotype, echoing previous findings that the canonical pathway may not be required for RME neurite A/P outgrowth (Figure 6C). To further address the biological relevance of CAM-1/DSH-1 binding, we made *dsh-1*;*cam-1*(null/weak) double mutants and found the double mutant phenotype is similar to *dsh-1* single, in consistence with the notion that these two are likely in a same pathway (Figure 5D). Furthermore, *cam-1(null)* results into much strong phenotype than either *cfz-2* or *mig-1* and *mig-1;cfz-2* double mimics *cam-1(null)*, suggesting CAM-1 plays a key role in transmitting CWN-2 signal to DSH-1 during RMED/V neurite outgrowth.

## Discussion

Proper neurite A/P guidance is important for establishing accurate three-dimensional neuronal connections in the nervous system. However, the underlying mechanisms have not been explored in detail. Through an unbiased genetic screen, we have revealed a particular Wnt-Frz/Ror-Dsh pathway that regulates neurite A/P growth in *C. elegans* GABAergic RMED/V motor neurons.

In *C. elegans*, several Wnts are known to function in nervous system development. *egl-20* is expressed in the posterior tail region and acts as a repellent cue to push HSN neuron migration forward [17]. *lin-44* participates in establishment of neuron cell polarity and this function is mediated by the Wnt receptor LIN-17 [15]. In the absence of *lin-44* and *lin-17*, the polarity of the PLM neuron is reversed. In addition, in *cwn-1;egl-20* double mutants, the polarity of the ALM neuron is reversed, suggesting that the precise combination of different Wnt pathway components may contribute to the specific functions of Wnts. *lin-44* and *lin-17* could also regulate the extension of neurites along the A/P axis through a β-catenin-dependent pathway [40]. Meanwhile, it has been reported that *lin-44*, *egl-20*, and *lin-17* also inhibit ectopic synapse formation in DA9 neurons [41]. Recently, CWN-2 has been linked to nerve ring organization, indicating that *cwn-2* is likely involved in nervous system development [16]. Our study provides additional evidence that CWN-2/Wnt and its downstream components are important for neural development.

One explanation for the observation that CWN-2 is essential for RMED/V neurite outgrowth while other Wnts are not, is that the specific functional requirement for CWN-2 is due to its intrinsic molecular features. However, in the absence of CWN-2, expression of *cwn-1*, *egl-20*, *lin-44* or *mom-2* in the nerve ring region partially rescued the neurite outgrowth defect of *cwn-2* mutant animals. In addition, while other Wnts (*cwn-1*, *egl-20*, *lin-44* and *mom-2*) are predominantly expressed in the posterior regions of the worm, *cwn-2* expression remains high in the anterior region, indicating that positioning Wnts at different places along the body axis could contribute to the functional specificity of Wnts.

Wnts have been implicated in the establishment of neuronal polarity. The lack of posterior RMED/V neurites in *cwn-2*, *dsh-1* and other mutants could result from loss of polarity instead of from outgrowth deficits. However, in *cwn-2* mutants, the processes around the nerve ring appear normal, indicating that cell polarity is at least partially maintained. Moreover, placing CWN-2 at the anterior-most region of the nerve ring with the *egl-17* promoter induces RMED neurites to grow forward, suggesting that CWN-2 likely functions in regulating neurite outgrowth. Consistent with this, ectopic expression of CWN-2 in a *cwn-2* mutant background with various promoters points to an attractive role of CWN-2 in neurite outgrowth.

Previous studies in *C. elegans* revealed that CAM-1 may antagonize Wnt activity [35], [36]. Furthermore, it has been shown that the extracellular domain (ECD) of CAM-1/Ror can bind Wnts in vitro. Thus it has been proposed that CAM-1 inhibits Wnt activity by sequestering Wnt ligands. In RMED/V motor neurons, however, CAM-1 apparently plays a positive role in mediating CWN-2/Wnt signals. First, *cam-1* mutants exhibit a similar phenotype to *cwn-2* single and *mig-1;cfz-2* double mutants. Second, the partial loss-of-function allele of *cam-1* synergistically enhances either the *cfz-2* or *mig-1* single mutant phenotype. Third, *cam-1* is expressed in and can function within RMED/V to guide neurite outgrowth. Together, the above data indicate that CAM-1 acts as a receptor with CFZ-2 or MIG-1, thus facilitating CWN-2 signal transduction. In addition, we found that neither CAM-1 intracellular domain nor DSH-1 has binding activity to CFZ-2 or MIG-1 intracellular domain (Figure 7C), implying that CAM-1 may play a central role in transducing CWN-2 signal to DSH-1 during RMED/V outgrowth. However, the facts that lacking of CAM-1 intracellular domain does not cause phenotype as strong as *dsh-1* null mutants and *cam-1* without kinase domain contains partial rescuing activity indicate that extracellular domain of CAM-1 also play a role in signal transduction, such as clustering other co-receptors, like CFZ-2 or MIG-1. In support of the role of CAM-1 in facilitating Wnt signaling, recent studies demonstrated that Wnts and CAM-1 work together to promote axon stability as well as to stabilize nerve ring organization [14], [16]. Why is CAM-1 needed in addition to both the CFZ-2 and MIG-1 receptors? Similar to Frz receptors, CAM-1/Ror has a CRD domain in the extra-cellular region, but unlike Frz receptors, CAM-1 only has a single transmembrane domain followed by a kinase domain. Although the kinase domain is not essential for CAM-1 activity, deletion or mutation of this domain compromised the Wnt signal, leading to a partial loss-of-function phenotype of *cam-1*. Thus, we suspect that different combinations of multiple Wnt receptors may contribute to the signal specificity of Wnt and to fine tuning of the Wnt signal. Although further investigation is required to test this speculation, a comparison can be made to the Netrin pathway: when UNC-40/DCC acts alone, it mediates Netrin-induced axon attraction, while with UNC-5, it leads axons away from the Netrin source [2], [42].

Out of three *dsh* genes, *dsh-1* is the only one which when mutated displays a similar phenotype to *cwn-2*, suggesting that the CWN-2 signal is largely mediated by DSH-1. When it goes to the downstream of DSH-1, we believe that CWN-2 signals do not go through the canonical pathway but instead act on the actin/microtubule cytoskeleton, because *unc-73/Trio*, *unc-34/enable*, *unc-14*, and *unc-76* mutant animals also display neurite outgrowth defects. Most importantly, mutants of two small GTPases (MIG-2/Rho and CED-10/Rac) exhibit class II mutant phenotypes, suggesting that they might act redundantly in regulating the cytoskeleton during neurite outgrowth. However, how Wnt signal is transduced from DSH-1 to the small GTPases, such as MIG-2/Rho and CED-10, is currently unknown. Cloning of other class I mutants may provide additional information.

Once axons grow out of the cell body, what kind of genetic program controls the length of those axons? Since ectopic expression of CWN-2 in the anterior nerve ring induces anterior neurite growth, we wondered whether elevating Wnt activity by over-expressing CWN-2 in wild-type animals could make RMED/V neurites grow longer. We tested other Wnts and found that none of them resulted in a neurite ‘over-shooting’ phenotype (data not shown). Therefore, the Wnt ligand level does not sufficient to determine the length of RMED/V posterior neurites. Through the screen, we identified a set of mutants with short or no posterior processes, but were unable to recover any mutants with neurites that were too long or incorrectly oriented (towards the anterior, for instance). One possible explanation is that genes regulating RMED/V neurite length or orientation choice are essential for viability. These mutants may be isolated in conditional screens. Another possibility is that genes controlling neurite length function redundantly. These genes may be identified through a gain-of-function analysis.

Given that four RME neurons with similar function are located in close proximity, why do RMED and RMEV extend neurites in response to CWN-2 while RMEL and RMER do not? Both CAM-1 and DSH-1 are expressed in all four RME neurons (Figure 5E and data not shown). Previously, we reported that in *ahr-1* mutants, all four RME neurons send out posterior processes [21], and we found that this phenotype is dependent on *cwn-2* (data not shown). AHR-1 is specifically expressed in RMEL/R but not RMED/V. Furthermore, ectopic expression of *ahr-1* in RMED/V neurons can result in loss of posterior processes, implying that *ahr-1* may act in potential Wnt-sensing neurons to antagonise the Wnt response. The targets of AHR-1 in the inhibition of Wnt signaling remain to be identified.

While the role of Wnts in regulating axon A/P guidance is well established, many questions remain. What is the specific combination of Wnt ligand, Wnt receptor, Dsh, and downstream signals in different circumstances? How does an individual neuron simultaneously sense the various guidance cues, including both D/V and A/P cues, present in the surrounding environment and thus decide which path it should take? How are the gradients of Wnt and other guidance cues established during development? How do they interact with each other? Answers to the above questions are certain to greatly advance our understanding of the principles of nervous system organization.

## Materials and Methods

### Strains and genetics

*C. elegans* were maintained on NGM plates under standard conditions [43]. *unc-30(ju54);juIs76* animals were treated with EMS and mutants with defective neurite outgrowth were isolated in the F2 generation. *juIs76* is an integrated transgenic line of *Punc-25::GFP* for labeling GABA motor neurons. A total of 10,000 mutagenized haploid genomes were screened and 18 mutants were recovered. *cwn-2(xd1)* was mapped to chromosome IV because of its linkage to *unc-30(ju54)*IV. *cam-1(xd13)* was mapped to chromosome II around +0.08 and complementation tests were performed between *cam-1(gm122)*, *cam-1(xd22)* and *cam-1(xd13)*. Alleles used in this study were listed in the Text S1.

### Molecular cloning and germline transformation

A *cwn-2* genomic DNA fragment containing 5kb promoter, coding region and 0.8kb 3′-UTR was amplified from N2 genomic DNA to perform the rescue experiment. The 5kb *cwn-2* promoter was cloned between the SphI and BamHI sites of *pSL1190* and *pPD95.77::mCherry* to yield *Pcwn-2-pSL1190* and *Pcwn-2::mCherry-pPD95.77*. cDNAs of *egl-20*, *lin-44*, *mom-2* and *cwn-1* were sub-cloned into *Pcwn-2-pSL1190*. To detect the expression patterns of Wnts, a fusion PCR approach was used to place *mCherry* downstream of various Wnt promoters. Fusion PCRs were also used to generate fragments for ectopic expression of *cwn-2* under different promoters (*myo-2*, *hlh-17*, *glc-2*, *egl-17* and *lin-11*) except *elt-2*. The expression patterns of these promoters were confirmed by *mCherry* reporters. To generate *Punc-25::DSH-1a*, *Punc-25::DSH-1b*, *Punc-25::CAM-1b* and *Punc-25::CAM-1b-YFP* constructs, full length *dsh-1a*, *dsh-1b*, and *cam-1b* cDNAs were cloned between the NheI and SacI/SalI sites after the *unc-25* promoter in plasmid *pSC325*. To examine the *cam-1* expression pattern, DNA sequences upstream of the *cam-1* start codon were amplified using PCR and then cloned into appropriate vectors. Transgenic animals were made by standard microinjection procedures.

### Image collection and phenotypic quantification

Except Figure 5E, all the RMED/V process images were taken from animals with an *unc-30(ju54);juIs76* background. At least three transgenic lines for each construct were examined for rescue activity and/or expression patterns. Fluorescence images were taken with compound microscope and confocal microscope. The phenotypic quantification was acquired using NIS-Elements BR 3.0 software.

### Yeast two-hybrid screen

The yeast transformation procedure was conducted using standard techniques. The intracellular domain (438aa) coding region of CAM-1b was fused with *pBTM116* (LexA DNA binding domain, Leu selection) as the bait to screen a *C. elegans* mixed-stage cDNA library. Yeast strains bearing test plasmids were replicated onto -Leu-Trp-His plates containing 3-Amino-1,2,4-Triazole (3AT; 5 mM and 10 mM) to test potential interactions. Positive clones were analyzed by DNA sequencing. A prey plasmid harboring full length DSH-1a was isolated from the screen. The intracellular domains of MIG-1(46aa) and CFZ-2 (61aa) were cloned into pBTM116 as the same way of CAM-1b. Fusion PCR was used to generate CAM-1b ICD deletion *pBTM116* constructs. A series of DSH-1a deletions constructs were made in an analogous manner to delete the DAX, PDZ and DEP domains.

## Supporting Information

Figure S1 The expression patterns of Wnt ligands in *C. elegans*. Top panels: fluorescence and bright field images of mCherry expressing embryos; middle and bottom panels: fluorescence and bright field images of adults (head to the left). (A and B) *Pcwn-1::mCherry* and *Pmom-2::mCherry* are robustly expressed in the tail and weakly expressed in the vulva and body wall muscles. (B and C) *Pegl-20::mCherry* and *Plin-44::mCherry* are mainly expressed in the tail during the embryonic and adult stages.
(0.36 MB TIF)

Figure S2 Quantification of RMED/V neurite phenotypes in different mutants. The phenotype of RMED and RMEV were evaluated separately. Schematic diagrams of the different posterior and anterior neurite phenotypes are shown at the top. (A) The rescue activity of the *cwn-2* genomic fragment in *cwn-2(xd1)*. (B) The phenotype of anterior extension in *Pegl-17::CWN-2*-rescued *cwn-2* mutants can be fully suppressed in wild type and partially suppressed in other mutants. (C) The rescue activity of the *Punc-25::DSH-1* transgenes in *dsh-1(xd5)*. (D) The rescuing activity of various *cam-1* constructs. *cam-1(gm122)* can't be suppressed by *Pcwn-2::CWN-2*.
(0.91 MB TIF)

Figure S3 Quantification of the rescue activity of other Wnts when driven by the *cwn-2* promoter. The DNA concentrations for injection are 1 ng/µl except *Pcwn-2::LIN-44* (2 ng/µl).
(0.35 MB TIF)

Figure S4 The CWN-2 expression levels in different genetic backgrounds by quantitative RT-PCR.
(0.79 MB TIF)

Figure S5 The rescuing activity of *cam-1* transgene driven by *hlh-17*, *unc-86*, or *unc-4* promoter.
(0.21 MB TIF)

Text S1 Alleles used in the study.
(0.04 MB DOC)
