# Supplementary figures and images for: A Wnt-Frz/Ror-Dsh Pathway Regulates Neurite Outgrowth in Caenorhabditis elegans
Source: PLoS Genet. 2010 Aug 12;6(8):e1001056. doi: 10.1371/journal.pgen.1001056 (PMC2920835; doi:10.1371/journal.pgen.1001056)

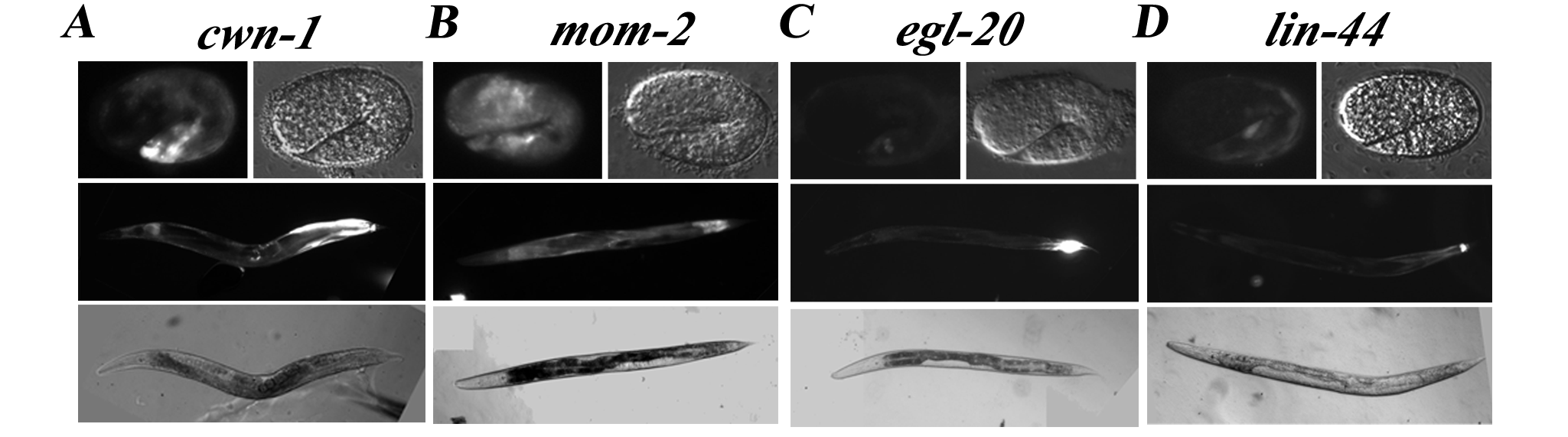

Supplement: Figure S1 — The expression patterns of Wnt ligands in C. elegans. Top panels: fluorescence and bright field images of mCherry expressing embryos; middle and bottom panels: fluorescence and bright field images of adults (head to the left). (A and B) Pcwn-1::mCherry and Pmom-2::mCherry are robustly expressed in the tail and weakly expressed in the vulva and body wall muscles. (B and C) Pegl-20::mCherry and Plin-44::mCherry are mainly expressed in the tail during the embryonic and adult stages. (0.36 MB TIF) [file pgen.1001056.s001.tif]

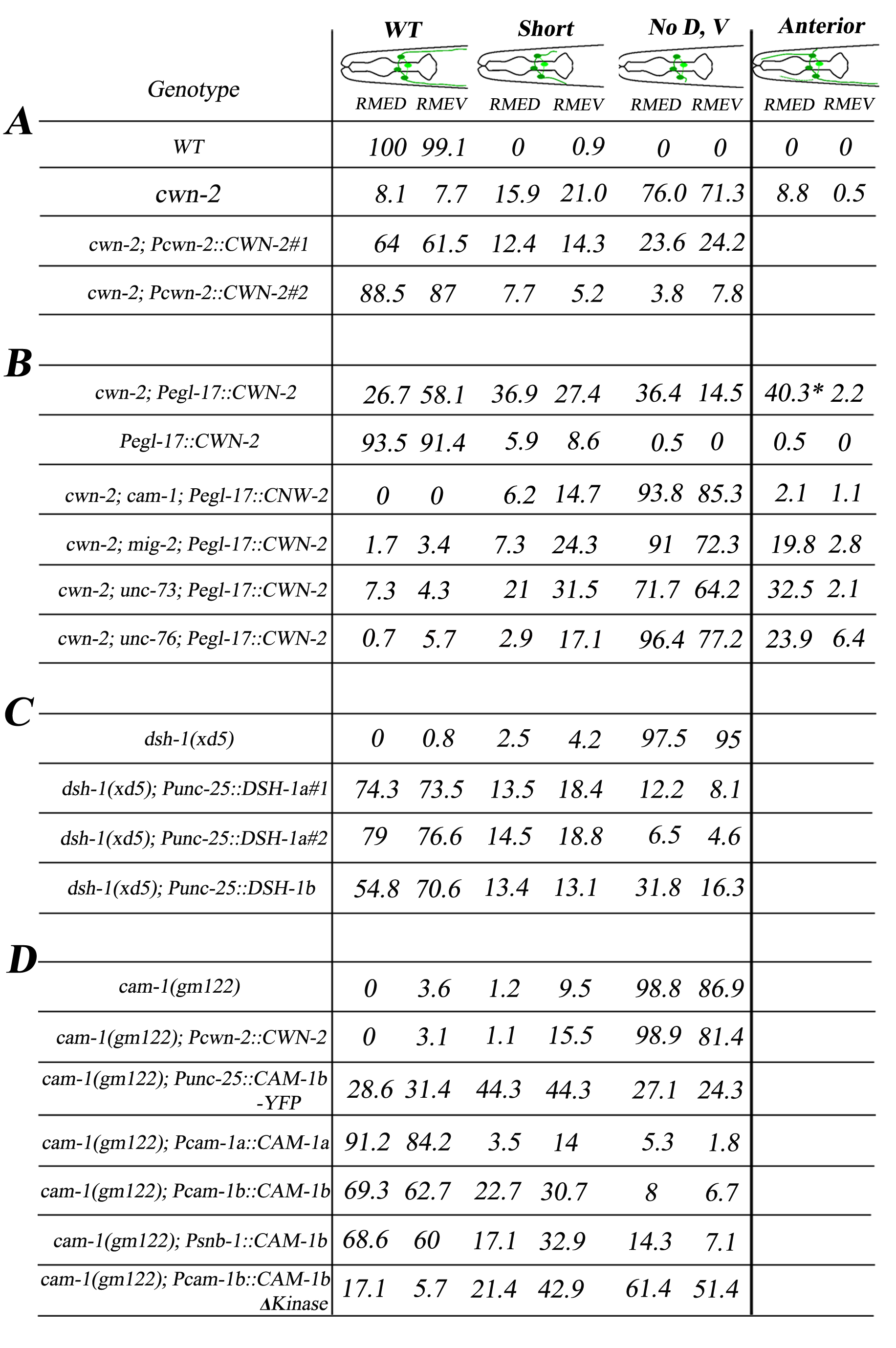

Supplement: Figure S2 — Quantification of RMED/V neurite phenotypes in different mutants. The phenotype of RMED and RMEV were evaluated separately. Schematic diagrams of the different posterior and anterior neurite phenotypes are shown at the top. (A) The rescue activity of the cwn-2 genomic fragment in cwn-2(xd1). (B) The phenotype of anterior extension in Pegl-17::CWN-2-rescued cwn-2 mutants can be fully suppressed in wild type and partially suppressed in other mutants. (C) The rescue activity of the Punc-25::DSH-1 transgenes in dsh-1(xd5). (D) The rescuing activity of various cam-1 constructs. cam-1(gm122) can't be suppressed by Pcwn-2::CWN-2. (0.91 MB TIF) [file pgen.1001056.s002.tif]

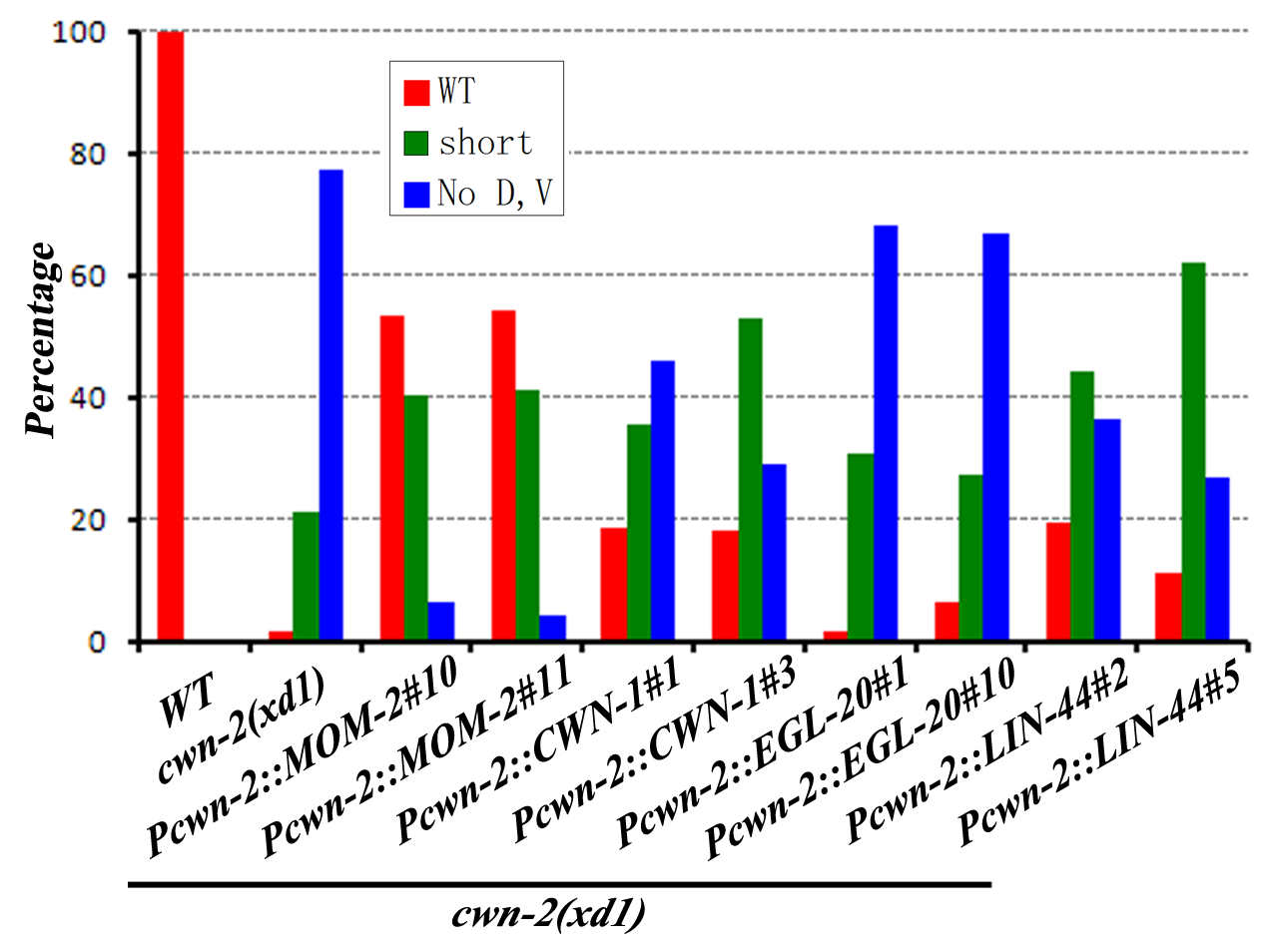

Supplement: Figure S3 — Quantification of the rescue activity of other Wnts when driven by the cwn-2 promoter. The DNA concentrations for injection are 1 ng/µl except Pcwn-2::LIN-44 (2 ng/µl). (0.35 MB TIF) [file pgen.1001056.s003.tif]

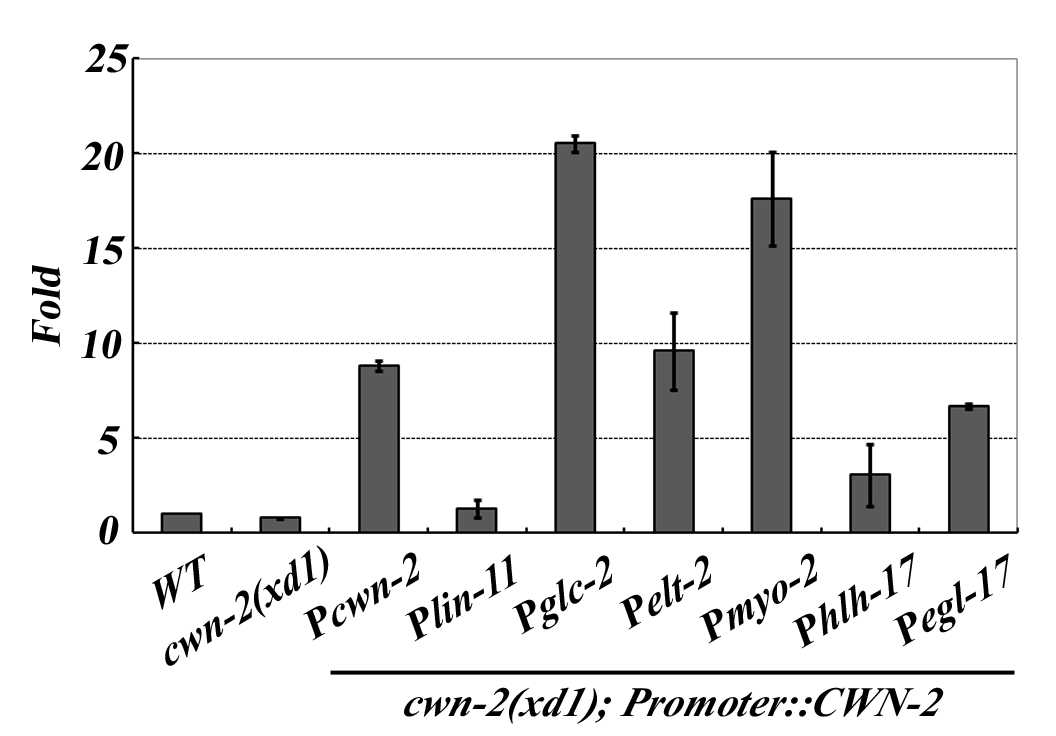

Supplement: Figure S4 — The CWN-2 expression levels in different genetic backgrounds by quantitative RT-PCR. (0.79 MB TIF) [file pgen.1001056.s004.tif]

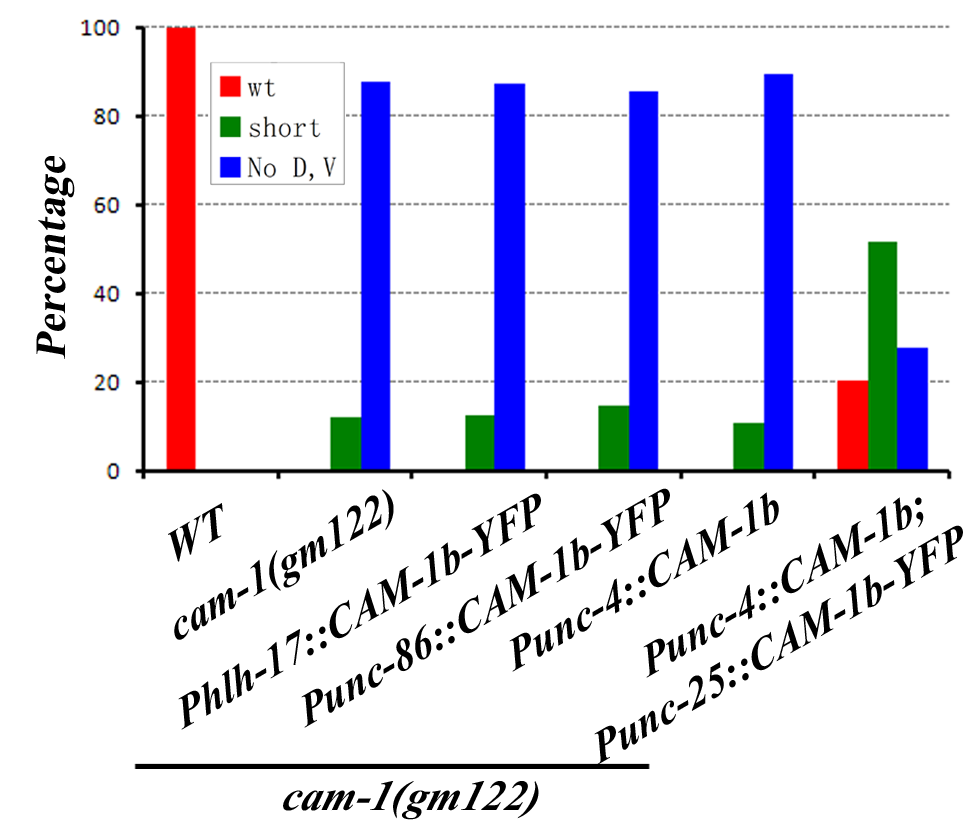

Supplement: Figure S5 — The rescuing activity of cam-1 transgene driven by hlh-17, unc-86, or unc-4 promoter. (0.21 MB TIF) [file pgen.1001056.s005.tif]
