# Supplementary material for: A Wnt-Frz/Ror-Dsh Pathway Regulates Neurite Outgrowth in Caenorhabditis elegans
Source: PLoS Genet. 2010 Aug 12;6(8):e1001056. doi: 10.1371/journal.pgen.1001056 (PMC2920835; doi:10.1371/journal.pgen.1001056)
Supplement: Text S1 — Alleles used in the study. (0.04 MB DOC) [file pgen.1001056.s006.doc]

Alleles used in the study.

I. *xdEx*

*xdEx1[Popt-3::mCherry,pRF4]*

*xdEx475[Pcwn-2::CWN-2cDNA,odr-1-RFP]*

*xdEx60[Pcwn-2::CWN-2genomic,pRF4]*

*xdEx61[Pcwn-2::CWN-2genomic,pRF4]*

*xdEx474[Pmyo-2::CWN-2genomic,odr-1-RFP]*

*xdEx524[Pglc-2::CWN-2genomic,odr-1-RFP]*

*xdEx467[Phlh-17::CWN-2genomic,odr-1-RFP]*

*xdEx543[Plin-11::CWN-2genomic,odr-1-RFP]*

*xdEx471[Pegl-17::CWN-2genomic,odr-1-RFP]*

*xdEx578[Pelt-2::CWN-2cDNA,odr-1-RFP]*

*xdEx62[Pcwn-1::mcherry,pRF4]*

*xdEx114[Pcwn-2::mcherry-pPD95.77,pRF4]*

*xdEx29[Pmom-2::mcherry,pRF4]*

*xdEx39[Plin-44::mcherry,pRF4]*

*xdEx52[Pegl-20::mcherry,pRF4]*

*xdEx137[Pmyo-2::mcherry,pRF4]*

*xdEx191[Pglc-2::mcherry,pRF4]*

*xdEx531[Phlh-17::mCherry,pRF4]*

*xdEx119[Plin-11::mcherry,pRF4]*

*xdEx530[Pegl-17::mCherry,pRF4]*

*xdEx562[Pelt-2::mCherry,pRF4]*

*xdEx40[Punc-25::DSH-1a,pRF4]*

*xdEx41[Punc-25::DSH-1a,pRF4]*

*xdEx608[Punc-25::DSH-1b,odr-1-RFP]*

*xdEx471[Pegl-17::CWN-2genomic,odr-1-RFP]*

*xdEX478[Pdsh-1a::mCherry,pRF4]*

*xdEx486[Pdsh-1b::mCherry,pRF4]*

*xdEx207(Pcam-1a::GFP, pRF4)*

*xdEx44(Pcam-1b::GFP, pRF4 )*

*xdEx88(Punc-25::CAM-1b, odr-1-RFP)*

*xdEx371(Punc-25::CAM-1b-YFP, odr-1-RFP)*

*xdEx343 (Punc-25::CAM-1b-YFP, Punc-4::cam1b, odr-1-RFP)*

*xdEx92(Pcam-1b::CAM-1b, odr-1-RFP)*

*xdEx202(Pcam-1a::CAM-1a, odr-1-RFP)*

*xdEx254(Psnb-1::CAM-1b, odr-1-RFP)*

*xdEx370(Punc-4:: CAM-1b, odr-1-RFP)*

*xdEx639(Phlh-17::CAM-1b-YFP, odr-1-RFP)*

*xdEx636 (Punc-86::CAM-1b-YFP, odr-1-RFP)*

*xdEx197(Pcam-1b:: CAM-1b△Kinase, odr-1-RFP)*

*xdEx249(Pcam-1b::CAM-1b△CRD, odr-1-RFP)*

*xdEx665(Pcwn-2::MOM-2, odr-1-RFP) line9 20ng/ul*

*xdEx685(Pcwn-2::MOM-2, odr-1-RFP) line10 20ng/ul*

*xdEx649(Pcwn-2::CWN-1, odr-1-RFP) line3 20ng/ul*

*xdEx652(Pcwn-2:: CWN-1, odr-1-RFP) line8 20ng/ul*

*xdEx688(Pcwn-2::EGL-20, odr-1-RFP) line2 20ng/ul*

*xdEx648(Pcwn-2::EGL-20, odr-1-RFP) line5 20ng/ul*

*xdEx718(Pcwn-2::MOM-2, odr-1-RFP) line10 1ng/ul*

*xdEx719(Pcwn-2::MOM-2, odr-1-RFP) line11 1ng/ul*

*xdEx720(Pcwn-2::CWN-1, odr-1-RFP) line1 1ng/ul*

*xdEx721(Pcwn-2::CWN-1, odr-1-RFP) line3 1ng/ul*

*xdEx722(Pcwn-2::EGL-20, odr-1-RFP) line1 1ng/ul*

*xdEx723(Pcwn-2::EGL-20, odr-1-RFP) line10 1ng/ul*

*xdEx674(Pcwn-2::LIN-44, odr-1-RFP) line2 2ng/ul*

*xdEx677(Pcwn-2::LIN-44, odr-1-RFP) line5 2ng/ul*

II. alleles

**LGI:** *lin-44(n1792), lin-11(n389), lin-17(n671), mig-1(e1787), mom-5(ne12), sys-1(q544), unc-14(e57)*

**LGII:** *juIs76[Punc-25-GFP, lin-15(+)], cwn-1(ok546), cam-1(gm122), cam-1(ks52), cam-1(xd13), cam-1(gm105), cam-1(xd22), dsh-1(xd5), dsh-1(ok1445), mig-14(ga62), vps-35(hu68), unc-73(e936)*

**LGIII:** *juIs73[Punc-25::GFP, lin-15(+)],wrm-1(ne1982)*

**LGIV :** *egl-20(n585), unc-30(ju54), cwn-2(ok895), cwn-2(xd1), unc-43(e408), rac-2(ok326), jnk-1(gk7), ced-10(n1993)*

**LGV:** *cfz-2(ok1201), unc-34(e566), unc-76(e911)*

**LGX:** *unc-6(ev400), slt-1(eh15), lin-18(e620), bar-1(ga80), vang-1(ok1142), mig-2(mu28)*
